# Diazepam causes sedative rather than anxiolytic effects in C57BL/6J mice

**DOI:** 10.1038/s41598-021-88599-5

**Published:** 2021-04-29

**Authors:** Marina Pádua-Reis, Diana Aline Nôga, Adriano B. L. Tort, Martina Blunder

**Affiliations:** 1 Behavioral Neurophysiology, Department of Neuroscience, Uppsala University, 75124 Uppsala, Sweden; 2 Brain Institute, Federal University of Rio Grande do Norte, Natal, RN 59056 Brazil

**Keywords:** Pharmacology, Experimental models of disease, Preclinical research

## Abstract

Diazepam has been broadly accepted as an anxiolytic drug and is often used as a positive control in behavioral experiments with mice. However, as opposed to this general assumption, the effect of diazepam on mouse behavior can be considered rather controversial from an evidence point of view. Here we revisit this issue by studying the effect of diazepam on a benchmark task in the preclinical anxiety literature: the elevated plus maze. We evaluated the minute-by-minute time-course of the diazepam effect along the 10 min of the task at three different doses (0.5, 1 and 2 mg/kg i.p. 30 min before the task) in female and male C57BL/6J mice. Furthermore, we contrasted the effects of diazepam with those of a selective serotoninergic reuptake inhibitor (paroxetine, 10 mg/kg i.p. 1 h before the task). Diazepam had no anxiolytic effect at any of the tested doses, and, at the highest dose, it impaired locomotor activity, likely due to sedation. Noteworthy, our results held true when examining male and female mice separately, when only examining the first 5 min of the task, and when animals were subjected to one hour of restrain-induced stress prior to diazepam treatment. In contrast, paroxetine significantly reduced anxiety-like behavior without inducing sedative effects. Our results therefore suggest that preclinical studies for screening new anxiolytic drugs should be cautious with diazepam use as a potential positive control.

## Introduction

Preclinical assessments are essential for understanding the biological basis of anxiety and are broadly used for screening new anxiolytic drugs^1^. Animal models for evaluating anxiety-like behavior are sensitive to clinically effective pharmacological treatments (predictive validity) and are based on a similar theoretical rationale underlying human behavior (construct validity)^2^. For example, the anxiety caused by an approach-avoidance conflict is common to both humans and rodents. Unfortunately, however, most of the drugs pointed out as anxiolytics by preclinical studies fail in having their effects corroborated by clinical trials^2–4^.

Diazepam (Valium®) is recommended for the treatment of recurrent convulsive seizures, for critical care sedation, and short-term relief of anxiety symptoms^5^. Like other benzodiazepines, diazepam increases the binding of endogenous gamma-aminobutyric acid (GABA) to its receptors, allosterically potentiating GABAergic signaling and promoting an overall depression in brain activity^6^. Diazepam side-effects, such as cognitive impairment, dependence, lethargy, falls, and even motor vehicle accidents are well documented since its launch in the early 1960s^7–9^. Until nowadays, however, this drug is overprescribed by clinicians and commonly used as a positive control in behavioral experiments with rodents for establishing the validity of new anxiolytic treatments^10,11^.

Nevertheless, the effects of diazepam on rodent anxiety-like behaviors are controversial. For instance, an influential review^12^ showed that from a total of 52 reviewed studies using the open field test in rodents, diazepam exhibited an anxiolytic effect in only 55.8% of them (29/52). In comparison, the remaining 23 studies (44.2%) found either the lack of effect (14 studies) or even the induction of anxiogenic-like behaviors (9 studies). Moreover, a recent meta-analysis revealed a publication bias in this literature, which tends to heavily favor the publication of positive results and omit negative findings^10^. The number of hypothesized missing data to correct the publication bias are noteworthy 102 experiments for the 386 analyzed ones^10^.

In the present work, we sought to revisit the effect of diazepam in a widely used paradigm for assessing anxiety-like behavior in preclinical studies: the elevated plus maze test^13^. We evaluated the minute-by-minute time-course of diazepam effect along the 10 min of the task at three different doses, in both female and male mice. Furthermore, we contrasted the effects of diazepam with those of a selective serotonergic reuptake inhibitor (paroxetine), and also tested the effects of these drugs in the open field test. As will be shown below, our findings add to the evidence that diazepam would not be anxiolytic in C57BL/6J mice. Therefore, we recommend caution when using it as a positive control in preclinical anxiety studies.

## Material and methods

### Animals

We used 116 female and 118 male adult C57BL/6J (Jackson Laboratory) mice (9–19 weeks old, 20–35 g) raised in the Uppsala University animal facilities. Animals were group-housed (up to five mice per cage) in transparent individual ventilated cages (35.5 × 17.5 cm, height 12.5 cm) containing wood-chip bedding, a paper house and two sheets of paper (Cellstoff, Papyrus) as enrichment. The cages were placed in temperature (19–21 °C) and humidity (65–70%) controlled cabinets in an animal room with a 12-h light/12-h dark cycle (lights on at 7 am). Throughout the experiments, the animals were maintained on standard mouse chow and water ad libitum*.* All animals were marked with ear tags for identification. Experiments were performed in the light phase and experimental groups were assembled using stratified randomization. All animal experiments were approved by the Uppsala Animal Ethical Committee (03686/2018 and 12149/2020) and followed the ARRIVE guidelines (Animal Research: Reporting of In Vivo Experiments) as well the Swedish Legislation on Animal Experimentation (Animal Welfare Act SFS 2009:303) and the European Union Directive on the Protection of Animals Used for Scientific Purposes (Directive 2010/63/EU).

### Drugs

Diazepam and paroxetine hydrochloride hemihydrate were obtained from Sigma-Aldrich, aliquoted upon arrival and diluted in vehicle (mixture of 10% DMSO and 5% TWEEN 80 in saline) before experiments. Diazepam (0.5, 1 and 2 mg/kg) was injected intraperitoneal (i.p.) 30 min prior to the behavioral tests, while paroxetine (10 mg/kg) was injected i.p. 1 h before. Drug doses were based on pilot experiments and previous studies^14–17^.

### Experimental cohorts

For all experiments, animals were randomly assigned to treatment groups according to the randomized block design^18^. Animals were divided into three experimental cohorts: (1) non-restrained animals treated with diazepam, (2) restrained animals treated with diazepam, and (3) non-restrained animals treated with paroxetine. For the first cohort, we used 50 female mice divided into two experimental batches of 23 and 27 animals, and 49 males in batches of 24 and 25 animals. The same animals were first exposed to the elevated plus maze and then to the open field, with an inter-session interval of at least two days to guarantee drug washout. A total of 6 female and 4 male mice were excluded from the elevated plus maze analysis due to exclusion criteria, which were either jumping from the apparatus, drug backflow during injections, or software tracking issues. The final number of animals in each group for the elevated plus maze was: vehicle = 23 (11 males and 12 females), diazepam 0.5 mg/kg = 25 (12 males and 13 females), diazepam 1 mg/kg = 21 (11 males and 10 females), diazepam 2 mg/kg = 20 (10 males and 10 females). A total of 1 male and 4 female mice were excluded from the open field analysis due to exclusion criteria, which were either drug backflow during injections, or software tracking issues. The final number of animals in each group for the open field was: vehicle = 24 (12 males and 12 females), diazepam 0.5 mg/kg = 23 (12 males and 11 females), diazepam 1 mg/kg = 24 (12 males and 12 females), diazepam 2 mg /kg = 23 (12 males and 11 females).

For the second cohort, we used 42 female mice divided into two experimental batches of 22 and 20 animals, and 45 males in batches of 24 and 21 animals. These animals were exposed to the elevated plus maze after being subjected to restraint-stress. A total of 2 female and 4 male mice were excluded from the analysis due to exclusion criteria. The final number of animals in each group was: vehicle = 21 (11 males and 10 females), diazepam 0.5 mg/kg = 19 (10 males and 9 females), diazepam 1 mg/kg = 21 (11 males and 10 females), diazepam 2 mg/kg = 20 (9 males and 11 females).

Finally, for the third cohort we used 24 female mice divided into two experimental batches of 16 and 8 animals, and 24 males in batches of 14 and 10 animals. The same animals were exposed to the elevated plus maze and then to the open field, with an inter-session interval of at least two days. A total of 4 males were excluded from the elevated plus maze analysis due to exclusion criteria. The final number of animals for the elevated plus maze session was: vehicle = 23 (11 males and 12 females), paroxetine 10 mg/kg = 21 (9 males and 12 females). No animal was excluded from the open field analysis: vehicle = 24 (12 males and 12 females), paroxetine 10 mg/kg = 24 (12 males and 12 females).

### Behavioral assessments

All animals were first left to acclimatize for at least 30 min in the same experimental room where they would be later subjected to the behavioral task. Male and female animals were tested in different time slots, with one sex being tested in the morning and the other in the afternoon. The allocation of sex per time window was balanced among batches, meaning in one batch females were tested in the morning, while in the next, they were tested in the afternoon. Mice were handled for five days prior to behavioral testing in order to avoid extra stress during experiments (n = 147), except for the subgroup of mice subjected to restraint-stress (n = 87), which did not undergo handling. On the experimental day, mice in this subgroup were restrained within a tube (length: 11.5 cm, diameter: 2.7 cm) for 1 h before i.p. injection of vehicle or diazepam^19^. The room lights were set to 150 lx for the elevated plus maze and 40 lx for the open field to stimulate exploratory behavior. For both behavioral tasks, already tested mice were placed in a different cage in order to not interfere with the behavior of non-tested animals. The behavioral apparatuses were thoroughly cleaned with a 10% ethanol solution between trials to reduce odors from previous subjects. Mouse behavior was recorded and quantified with the EthoVision XT15 software (Noldus); behavioral data were subsequently exported to Matlab® for further analyses.

### Elevated plus maze

The elevated plus maze we used consists of a cross-shaped plastic apparatus, elevated 50 cm from the floor, with two opposite open arms and two opposite enclosed arms. The floor of the arms was made of gray plastic, 37 cm long and 5.4 cm wide and connected by a central platform of 5.5 × 5.5 cm. Gray plastic walls of 15 cm height surrounded the enclosed arms. At trial start, mice were placed in the central zone of the maze, facing the same open arm and were allowed to freely explore the apparatus for 10 min. Of note, since several studies using the elevated plus maze have analyzed animals after either 5^13,20,21^ or 10^22–27^ min of maze exploration, we opted for a total of 10 min of task duration to be able to provide results for both these time points. As anxiety-like metrics^13,20,28^, we measured the number of entries in the open arms (scored when the center point of the body crossed into the open arm zone) and the percentage of time spent in the open arms omitting the central zone, that is, the percentage was calculated as 100*time in open arms/(time in open arms + *time in closed arms*). As metrics of exploratory behavior, we measured the time animals spent moving (scored when the body center point changed its location with a speed ≥ 2 cm/s), and the time spent in stretched-attend posture, defined to occur when the animal stretches its body without locomoting. The latter is usually considered as a risk-assessment behavior^29–31^.

### Open field

We employed a circular open arena (with a 50-cm diameter and a 60-cm high wall) made out of gray plastic. Mice were put in the center of the field at trial start, and allowed to freely explore the arena for 10 min. We measured the time animals spent moving (speed ≥ 2 cm/s), the total distance traveled, and the number of entries and time spent in the center zone (defined as the circular region encompassed by half the diameter).

### Data analysis

The behavioral metrics were obtained for every 1 min of the respective task duration. Cumulative curves along the 10-min session were then computed for each metric by summing all counts from past time bins to the current one. The exception was the percentage of time in the open arms, of which the cumulative curve was obtained by dividing the sum of the time spent in the open arms up to the time bin by the total time in open and closed arms at the end of the session. To allow for comparison with other studies, we provide bar graphs and statistical analyses for the behavioral metrics computed at 5 and 10 min of task duration for both the elevated plus maze and the open field. In the main figures, data for males and females are shown combined; supplementary figures show results separately for male and female mice (Figures S1–S5).

### Statistical analysis

Bar plots depict mean ± SD over animals. Differences between groups were assessed for statistical significance by either two-sample t-tests or one-way ANOVA followed by Tukey’s post hoc test. We set alpha = 0.05 to denote statistical significance.

## Results

The elevated plus maze paradigm has been classically and widely used to screen for anxiolytic drugs^28,32^. The maze is elevated from the ground and consists of two arms surrounded by walls (the “closed” arms) and two “open” arms. The rationale is that mice are assumed to feel safer in the closed arms, while excursions to the open arms would be dictated by a conflict between their natural drive for exploration and the avoidance of an aversive zone (i.e., brighter lit than the closed arms, open to potential predators, smoother surface, elevated from the ground). Open arm exploration is thus taken as a risk-assessment behavior and is associated with a lower anxiety-like state. In operational terms, the number of entries in the open arms and/or the proportion of time spent in open arms are usually taken as metrics for assessing anxiety-like behavior. That is, the higher these metrics, the lower the anxiety profile; conversely, the more time the animal spends in the closed arms, the more anxious it is assumed to be.

**Figure 1:**
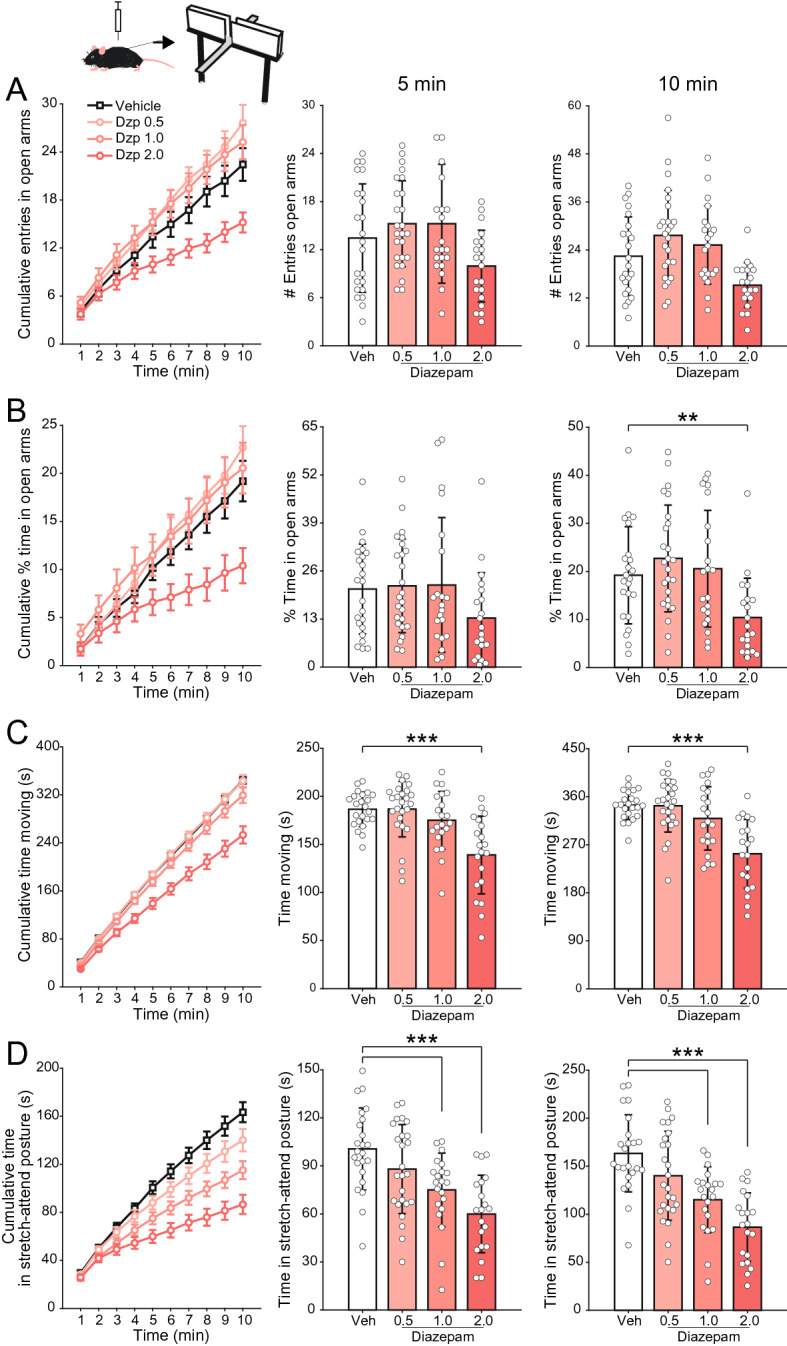
Lack of anxiolytic effect of diazepam in the elevated plus maze. (**A**) (Left) Cumulative number of entries in the open arms along the 10-min session. (Middle and right) Mean (± SD) number of open arm entries after 5 (middle) and 10 min (right). White circles show data for individual animals. Animals were injected i.p. with vehicle or three doses of diazepam (0.5, 1.0 and 2.0 mg/kg) thirty minutes prior to behavior testing, as labeled. (**B**–**D**) Panels show the same as in (**A**), but for the percentage of the time spent in the open arms (**B**), and the total time animals spent moving (**C**) or in stretch-attend posture (**D**). No anxiolytic effect of diazepam is found in any of the doses, as inferred by no increase in the metrics shown in (**A**) and (**B**) (on the contrary, the highest diazepam dose decreased open arm exploration). Notice further that diazepam induces both a reduction in locomotor activity (**C**) and risk assessment behaviors (**D**). **p < 0.001, ***p < 0.0001, one-way ANOVA followed by Tukey’s post hoc test.

We started by investigating the effect of three doses of diazepam (0.5, 1, and 2 mg/kg) in C57BL/6J mice subjected to a 10-min session in the elevated plus maze apparatus (n = 89 mice in this cohort, see Material and Methods). Diazepam or vehicle were injected i.p. 30 min prior to the behavior test. As shown in Fig. 1, mice treated with diazepam did not exhibit a higher number of entries in the open arms (Fig. 1A), nor spent a higher percentage of time in the open arms (Fig. 1B). Therefore, in our experiments, diazepam was clearly not anxiolytic in the elevated plus maze paradigm for any of the tested doses. Actually, we even found a potential anxiogenic effect of diazepam at the highest dose (2 mg/kg), in which animals spent less time exploring the open arms (Fig. 1B; % time in open arms, 10 min: F(3,85) = 5.57, p = 0.0015, one-way ANOVA followed by Tukey’s post hoc test), though at this same dose diazepam also reduced locomotion (Fig. 1C, 5 min: F(3,85) = 11.93, p < 10^–5^; 10 min: F(3,85) = 14.70, p < 10^–7^) . Moreover, diazepam dose-dependently reduced the time animals spent in stretch-attend posture (Fig. 1D, 5 min: F(3,85) = 14.82, p < 10^–7^; 10 min: F(3,85) = 14.70, p < 10^–7^). Notice that the latter finding shows that all diazepam doses were capable of influencing mouse behavior, thus the lack of an anxiolytic effect is not due to a lack of a drug effect.

**Figure 2:**
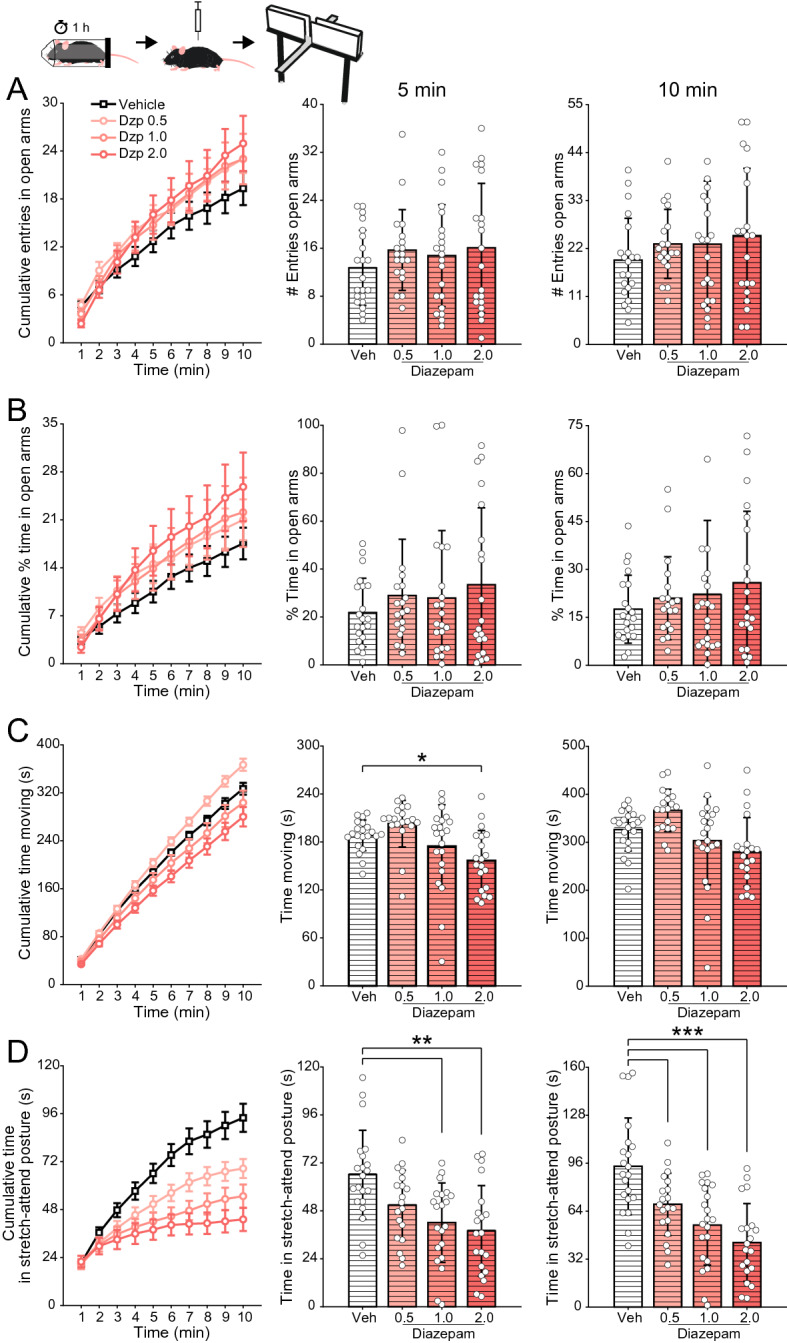
Lack of anxiolytic effect of diazepam in restraint-stressed animals. (**A**) (Left) Cumulative number of entries in the open arms of the maze along the 10-min session. (Middle and right) Mean (± SD) number of open arm entries after 5 (middle) and 10 min (right). White circles show data for individual animals. Animals were subjected to 1-h restraint-stress and then injected i.p. with vehicle or three doses of diazepam (0.5, 1.0 and 2.0 mg/kg) thirty minutes prior to behavior testing, as labeled. (**B**–**D**) Panels show the same as in (**A**), but for the percentage of the time spent in the open arms (**B**), and the total time animals spent moving (**C**) or in stretch-attend posture (**D**). Diazepam exhibited no statistically significant anxiolytic effect in this protocol, nor influenced locomotor activity. As in non-restrained animals (Fig. 1D), diazepam dose-dependently reduced the number of risk assessment behavior shown as reduced time in stretch-attend posture. *p < 0.01, **p < 0.001, ***p < 0.0001, one-way ANOVA followed by Tukey’s post hoc test. In A and B, outliers above the y-axis limit are not shown.

Previous studies have shown that an anxiolytic effect is more likely to be observed in stressed animals^33,34^. Therefore, we next devised a second cohort (n = 81 animals) to test whether diazepam is anxiolytic in animals subjected to restraint-stress before being tested in the elevated plus maze (see Materials and Methods for details). In addition to the restraint-stress, these animals were not previously habituated to the experimenter (i.e., manually handled). As shown in Fig. 2, under this protocol diazepam-treated animals did not statistically differ from vehicle-treated animals for the anxiety-related metrics (Fig. 2A,B). We note that even though their group means appeared to be dose-dependently higher upon visual inspection (Fig. 2A,B), this was mainly due to the presence of a few outliers. At the group level, due to the large variability, this visual tendency was far from reaching statistical significance (entries in open arms, 5 min: F(3,77) = 0.67, p = 0.57; 10 min: F(3,77) = 0.75, p = 0.52; % time in open arms, 5 min: F(3,77) = 0.73, p = 0.54; 10 min: F(3,77) = 0.72, p = 0.55). Regarding the other locomotor metrics, diazepam affected the amount of time animals spent moving during the first 5 min (Fig. 2C; F(3,77) = 5.64, p < 0.01), and, similar to non-restraint animals, it dose-dependently reduced stretch-attend postures (Fig. 2D, 5 min: F(3,77) = 7.47, p < 0.001; 10 min: F(3,77) = 13.65, p < 10^–6^).

**Figure 3:**
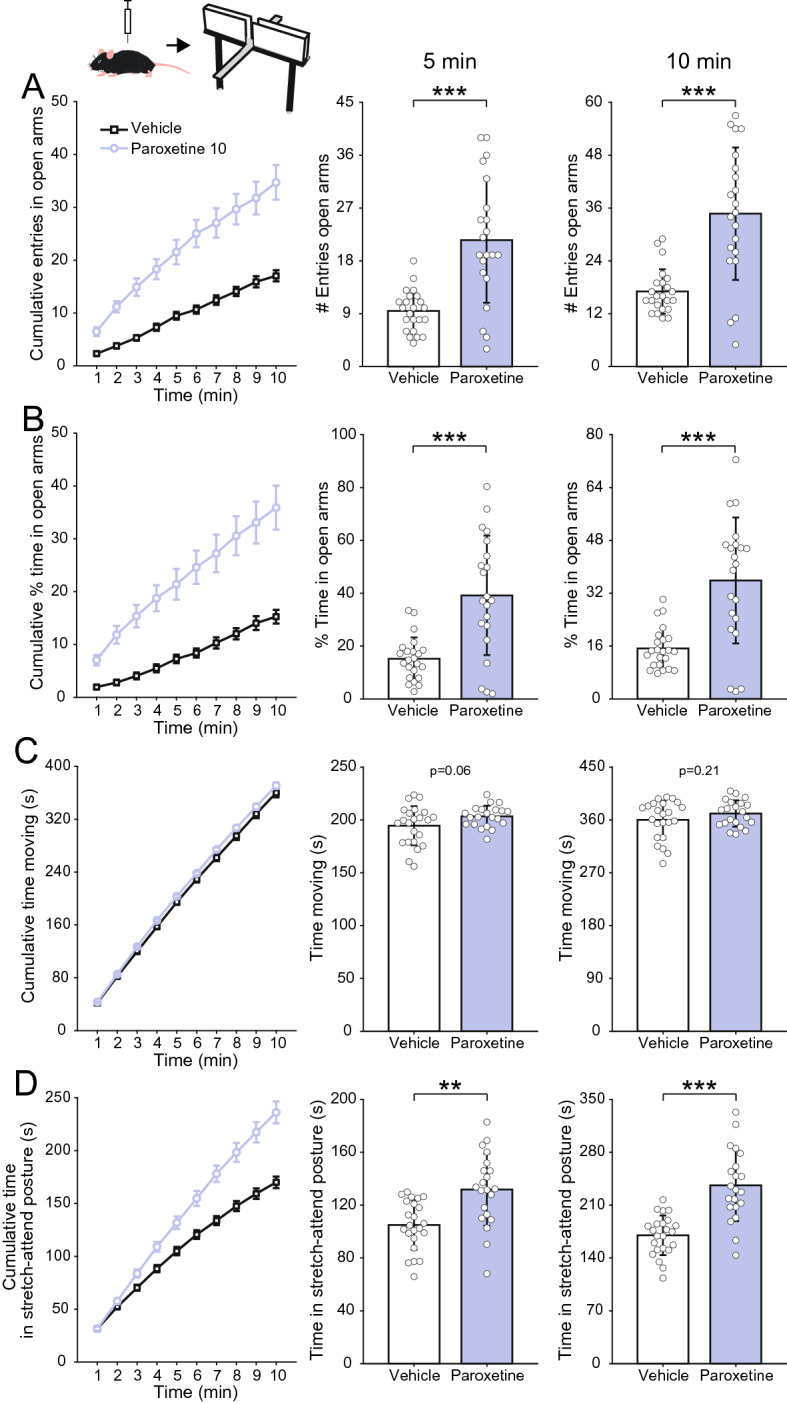
Anxiolytic effects of paroxetine in the elevated plus maze. (**A**) (Left) Cumulative number of entries in the open arms of the maze along the 10-min session. (Middle and right) Mean (± SD) number of open arm entries after 5 (middle) and 10 min (right). White circles show data for individual animals. Animals were treated with vehicle or paroxetine (10 mg/kg) 1 h prior to behavioral testing. (**B**–**D**) Panels show the same as in (**A**), but for the percentage of the time spent in the open arms (**B**), and the total time animals spent moving (**C**) or in stretch-attend posture (**D**). Paroxetine-treated animals exhibited a pronounced anxiolytic-like behavioral profile along with no change in locomotor activity and an increase in risk assessment behavior shown as increased time in stretch-attend posture. **p < 0.001, ***p < 0.0001, two-sample t-test.

We next sought to check for the predictive validity of the elevated plus maze test in screening for anxiety-modulating drugs. To that end, we devised a third cohort (n = 44 animals) using the selective serotonin reuptake inhibitor paroxetine (10 mg/kg) as a positive control drug. As shown in Fig. 3, our results largely confirmed the role of selective serotonin reuptake inhibitors in reducing anxiety-like behaviors^35,36^. Namely, paroxetine-treated animals exhibited both a much higher number of entries (Fig. 3A, 5 min: t(42) = − 5.13, p < 10^–5^; 10 min: t(42) =  − 5.32, p < 10^–5^, two-sample t-tests) and a higher percentage time spent in the open arms (Fig. 3B, 5 min: t(42) =  − 4.77, p < 0.0001; 10 min: t(42) =  − 4.92, p < 0.0001). Paroxetine seemed to slightly increase the time animals spent moving (Fig. 3C), but this effect was not statistically significant (5 min: t(42) =  − 1.95, p = 0.06; 10 min: t(42) =  − 1.28, p = 0.21). Noteworthy, paroxetine significantly increased time in stretch-attend posture (Fig. 3D, 5 min: t(42) =  − 3.82, p < 0.001; 10 min: t(42) =  − 5.79, p < 10^–6^).

**Figure 4:**
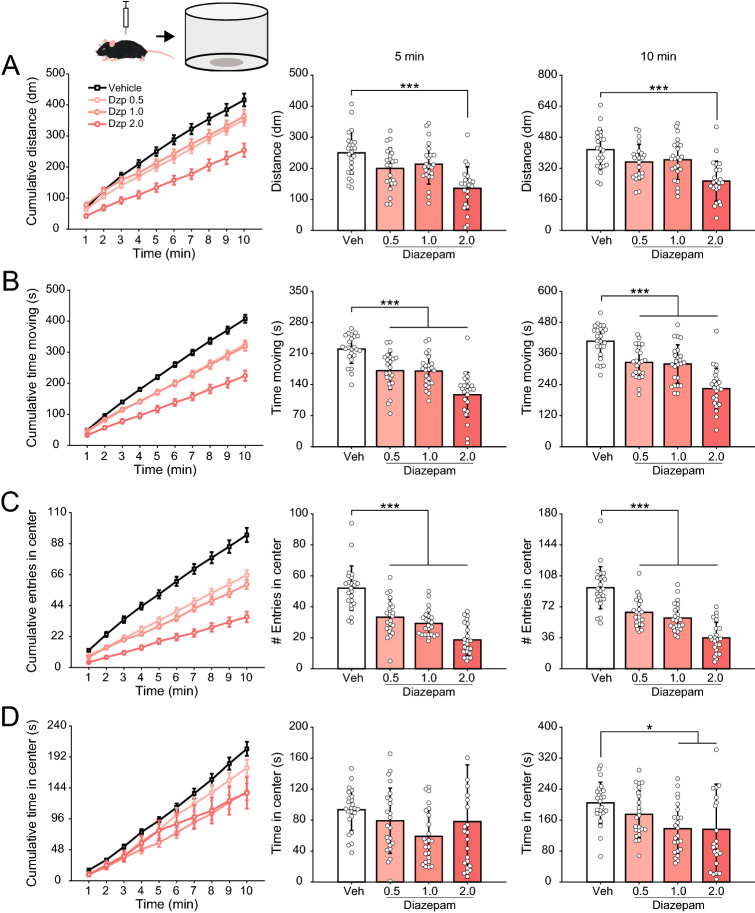
Diazepam effects in the open field. (**A**) (Left) Cumulative distance moved in the open field. (Middle and right) Mean (± SD) distance after 5 (middle) and 10 min (right). White circles show data for individual animals. Animals were injected i.p. with vehicle or three doses of diazepam (0.5, 1.0 and 2.0 mg/kg) thirty minutes prior to behavior testing, as labeled. (**B**–**D**) As before, but for the time spent moving (**B**), number of entries in the center zone (**C**) and time spent there (**D**). Diazepam reduced locomotor activity and the number of entries in the center zone. *p < 0.01, ***p < 0.0001, one-way ANOVA followed by Tukey’s post hoc test.

**Figure 5:**
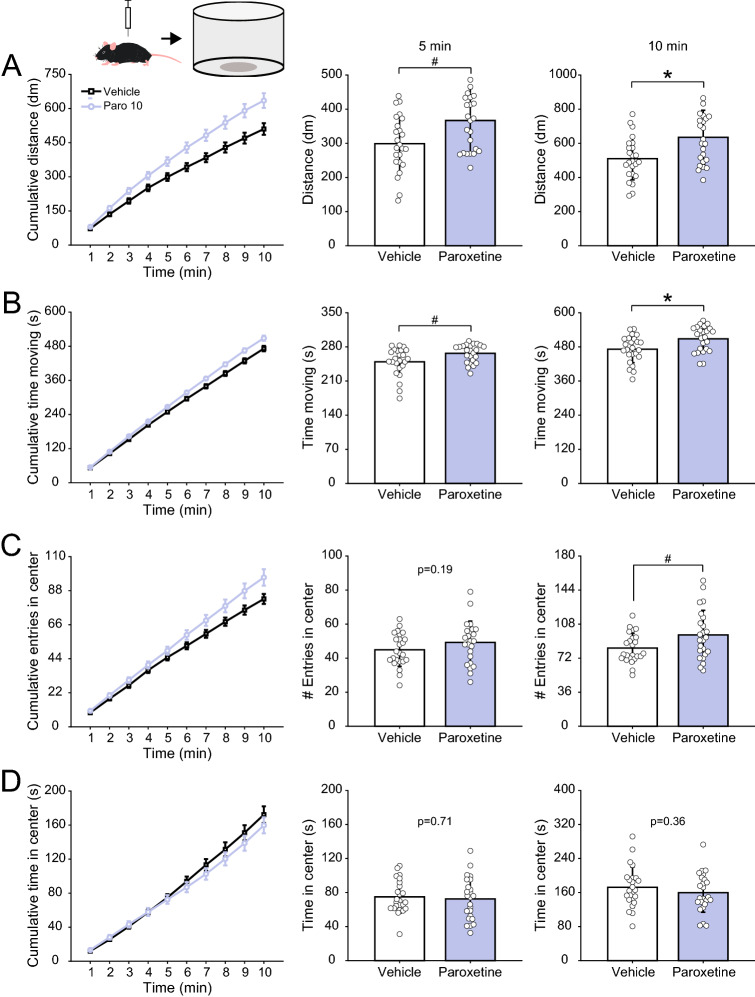
Paroxetine effects in the open field. (**A**) (Left) Cumulative distance moved in the open field. (Middle and right) Mean (± SD) distance after 5 (middle) and 10 min (right). White circles show data for individual animals. Animals were treated with vehicle or paroxetine (10 mg/kg) 1 h prior to behavioral testing. (**B**–**D**) As before, but for the time spent moving (**B**), number of entries in the center zone (**C**) and time spent there (**D**). Paroxetine increased locomotor activity and did not majorly influence the exploration of the center zone. ^#^p < 0.05, *p < 0.01, two-sample t-test.

Since the elevated plus maze results indicated that the drugs affect not only anxiety-related metrics but also locomotor activity, we next evaluated the effect of diazepam and paroxetine in mice exploring an open field arena. Consistent with the findings above, diazepam strongly reduced the time animals spent moving at all doses tested (Fig. 4B, 5 min: F(3,90) = 24.97, p < 10^–11^; 10 min: F(3,90) = 26.96, p < 10^–11^), and, at the highest dose, it decreased the total traveled distance (Fig. 4A, 5 min: F(3,90) = 11.55, p < 10^–5^; 10 min: F(3,90) = 10.85, p < 10^–5^). Of note, diazepam at all doses also decreased the number of entries in the open field center (Fig. 4C, 5 min: F(3,90) = 35.50, p < 10^–14^; 10 min: F(3,90) = 37.03, p < 10^–14^), and, at the doses of 1 and 2 mg/kg, it also decreased the time spent in the center for the whole 10 min session (Fig. 4D, F(3,90) = 4.26, p = 0.0073), which could potentially denote increased anxiety. On the other hand, paroxetine increased both total traveled distance (Fig. 5A, 5 min: t(46) =  − 2.67, p = 0.01; 10 min: t(46) =  − 3.04, p < 0.01) and the time spent moving (Fig. 5B, 5 min: t(46) =  − 2.47, p = 0.017; 10 min: t(46) =  − 2.74, p < 0.01) and slightly increased the number of entries in the center zone for the whole 10 min session (Fig. 5C right; t(46) =  − 2.25, p = 0.03), but otherwise it did not affect the center metrics (Fig. 5C,D).

In all, our results show that diazepam has no anxiolytic effect in C57BL/6J mice tested in the elevated plus maze and also that diazepam impairs locomotor activities in both the elevated plus maze and open field arenas, likely due to sedation. In contrast, paroxetine greatly reduces anxiety-like behavior without inducing sedative effects. Noteworthy, our results hold true when examining male and female mice separately in the elevated plus maze and the open field tests (Figures S1–S5).

## Discussion

In the present study, we re-evaluated the action of diazepam in two of the most commonly used tests for the screening of anxiolytic drugs in the preclinical literature: the elevated plus maze and the open field. To that end, we have studied both male and female C57BL/6J mice (234 animals in total) in well-powered cohorts (sample sizes usually > 20 animals per dose group). We have also investigated whether diazepam would differentially affect stressed animals. Although many are likely to consider the replication initiative intended here as not necessary given that both behavioral tests are well consolidated in the literature with diazepam as a classical positive control for them, our results challenge this widespread assumption. Namely, using a large sample of mice, we found no anxiolytic effect of diazepam in any of the studied doses in the elevated plus maze paradigm (Fig. 1A,B). Actually, if anything, diazepam at the highest dose (2 mg/kg) led to a potential anxiogenic profile in which animals explored less the open arms (Fig. 1A,B), though, at this same dose, diazepam also impaired locomotion (Fig. 1C). Noteworthy, diazepam dose-dependently reduced the time spent in stretch-attend postures (Fig. 1D). Further below we discuss the interpretation of such effect in more detail; in any case, for now we note that this result constitutes evidence that all tested diazepam doses worked properly; that is, the lack of a greater exploration of the open arms cannot be attributed to a lack of drug effect (i.e., to experimental issues).

The picture was not much different when we subjected animals to restraint-induced stress before diazepam treatment and elevated plus maze exploration (Fig. 2). The rationale for this protocol was to increase the basal levels of anxiety displayed by the animals, since previous studies have shown that anxiolytic drugs would particularly show their effects during heightened anxiety states, such as the one induced by stress^33,34^. These studies directly compared restrained and non-restrained animals and observed a decrease in open arm exploration in the former. However, other studies did not find any difference in open arm exploration after restraining^37,38^. Such a direct comparison is unfortunately not warranted in our dataset, at least not in a confident way, since our restrained and non-restrained animals pertained to different cohorts. That said, by performing straight comparisons, we could only see a slight reduction in open arm exploration. Nevertheless, the restraining protocol seemed to be effective in changing animal behavior through the stretch-attend posture: animals subjected to restraint-stress seems to spend less time displaying this posture (cf. Figs. 1D and 2D). At any event, for the cohort of restraint-stressed animals, the profile of open arm exploration did not differ between diazepam-treated animals and controls (Fig. 2A,B), although diazepam further decreased the stretch-attend postures dose-dependently (Fig. 2D). Curiously, we did not observe a tendency towards a potential anxiogenic profile for the highest diazepam dose (2 mg/kg) in restraint-stressed animals, a result that differed from non-stressed mice (cf. Figs. 1 and 2).

In the third cohort of animals, we sought to replicate the anxiolytic effect of paroxetine, another drug also typically used as positive control in the elevated plus maze task and which has a different mechanism of action from diazepam^15,39–41^. The results largely corroborated the literature by showing a much greater exploration of open arms by paroxetine-treated animals when compared to vehicle-treated animals (Fig. 3A,B). Of note, this constitutes solid evidence for the suitability of our elevated plus maze setup in identifying anxiolytic drugs, once again discarding experimental errors as possible reasons for the lack of diazepam effect in the task. Interestingly, paroxetine also exhibited contrasting effects with diazepam regarding the time spent in stretch-attend postures, which was significantly increased (Fig. 3C). In all, our results suggest that selective serotoninergic reuptake inhibitors such as paroxetine are much more effective than benzodiazepines like diazepam in reducing anxiolytic-like behavior in C57BL/6J mice exposed to the elevated plus maze.

Of note, in humans, selective serotonin reuptake inhibitors usually only show anxiolytic or antidepressant effects after two to three weeks and, moreover, sometimes they have an acute anxiogenic response^45^. This contrasts with our results showing an acute anxiolytic effect of paroxetine. Nevertheless, several studies in rodents using the elevated plus maze and other tasks that evaluate anxiety-like behavior are consistent with ours, in which they show an anxiolytic action following acute administration of serotonin reuptake inhibitors^15,41,42^. And a similar situation is seen in tasks used for antidepressant screening^41,43–45^. Thus, we believe the contrast between the acute effects of paroxetine in rodents and humans does not invalidate the use of acute paroxetine as a positive control in the elevated plus maze.

Finally, we further contrasted the effects of diazepam and paroxetine in the open field test. The analysis of locomotor activity showed a dose-dependent sedative effect by diazepam, in which animals traveled shorter distances and spent less time moving (Fig. 4). Paroxetine, on the other hand, significantly increased both metrics (Fig. 5). This indicates that the anxiolytic effect of paroxetine, as inferred by the elevated plus maze results, is not without changes in locomotor activity, though, curiously, in the elevated plus maze test itself paroxetine had no such effect (Fig. 3C). Regarding exploration of the center zone, a typical anxiety metric in open field test^12^, we found a decrease of center exploration in diazepam-treated animals. While the latter result could be due to sedation—as opposed to a true anxiogenic action—it certainly does not support the use of diazepam as a positive anxiolytic control in this task. In this regard, a literature review^12^ showed that around half (44%) of the studies examining the effect of diazepam in the open field test found no evidence for it, thus consistent with the current findings.

Of note, similar results were obtained when examining male and female mice separately (Figures S1–S5), thus our main conclusion of lack of support for diazepam as a positive anxiolytic control in these tasks do not depend on sex. It may, however, depend on the mouse strain as previous studies have shown strain differences regarding drug effect on anxiety-like behavior^14,16,46,47^. Nevertheless, it is also important to highlight that others have reported an anxiolytic effect of diazepam in C57BL/6 mice exposed to the elevated plus maze, both when specifically testing the effects of this drug^14,47,48^, or when using it as a positive control^49,50^. The distinct results from the ones obtained here may be due to differences in mouse age^14,47^, apparatus characteristics such as floor texture, arm length and transparency^14,47,49^, light settings^48,50^, reduced number of subjects^14,49,50^, and light period^48^ used in the experiments. Moreover, a recent meta-analysis of another test that measures anxiety-like behavior, the marble burying test, showed a strong effect size when comparing diazepam to vehicle^42^ (but also see ref.^51^ for a discussion about marble burying and anxiety).

Both drugs influenced the time spent in stretch-attend postures, which are believed to reflect the ambivalence between avoidance and approach towards a novel and potentially aversive space, that is, a conflict of fear versus curiosity^30^. Consistent with our findings, previous studies have reported that diazepam reduces the time animals spend in this posture^52,53^. However, the authors interpreted this result as indicative of a reduction in anxiety levels, while we have a different perspective on it. Namely, we hypothesize that stretch-attend postures would be instead related to exploratory behavior. The reason for such interpretation is threefold: first, the highest diazepam dose (2 mg/kg), which causes the greatest decrease in such postures (Fig. 1D), is associated with the highest sedation and therefore less exploration (Fig. 4); second, restraint-stressed animals tend to exhibit fewer stretch-attend postures (cf. Figs. 1D and 2D), and they presumably have greater, not lower, anxiety levels; finally, paroxetine, which exhibited a clear anxiolytic profile (Fig. 3), actually increased the time spent in such postures. In all, these results indicate that when animals stretch and attend, they reflect a drive towards exploration.

The challenge to be overcome by preclinical studies on anxiety is multifactorial. It involves questioning classical behavior assessment adequacy, the understanding of animal models specificities and, last but not least, the use of appropriate positive controls. We certainly do not want to imply with the current results that diazepam should be discarded as an anxiolytic drug. However, what we do would like to call attention to are some of the common difficulties in preclinical research, which may ultimately underlie the lack of translatability. In this regard, when controls do not work, this tends to be commonly attributed to errors in the experimental setup or mistakes by the experimenter. Young researchers are especially vulnerable since they are more likely to be pressured to show they are able to reproduce classical control results to their supervisors. In contrast, questioning the validity of the control itself is a seldom practice^54,55^. We finish by recommending other researchers that are also unable to reproduce “established” results to publish their failed attempts; otherwise, the selective publication of positive results will end by biasing the literature and poisoning the scientific endeavor. Failed replications, as long as they were performed with an adequate sample size and under valid and reliable experimental conditions, should not be put into the drawer.

## Supplementary Information

Supplementary Information

## Data Availability

The datasets generated during the current study are available from the corresponding author on reasonable request.
