# Supplementary material for: Diazepam causes sedative rather than anxiolytic effects in C57BL/6J mice
Source: Sci Rep. 2021 Apr 29;11:9335. doi: 10.1038/s41598-021-88599-5 (PMC8085115; doi:10.1038/s41598-021-88599-5)
Supplement: Supplementary file 1 — Supplementary Information [file 41598_2021_88599_MOESM1_ESM.pdf]

Diazepam causes sedative rather than anxiolytic effects in C57BL/6J mice

Marina Pádua-Reis*, Diana Aline Nôga*, Adriano BL Tort, Martina Blunder

Supplementary Material

Content: 5 Supplementary Figures + Figure Legends

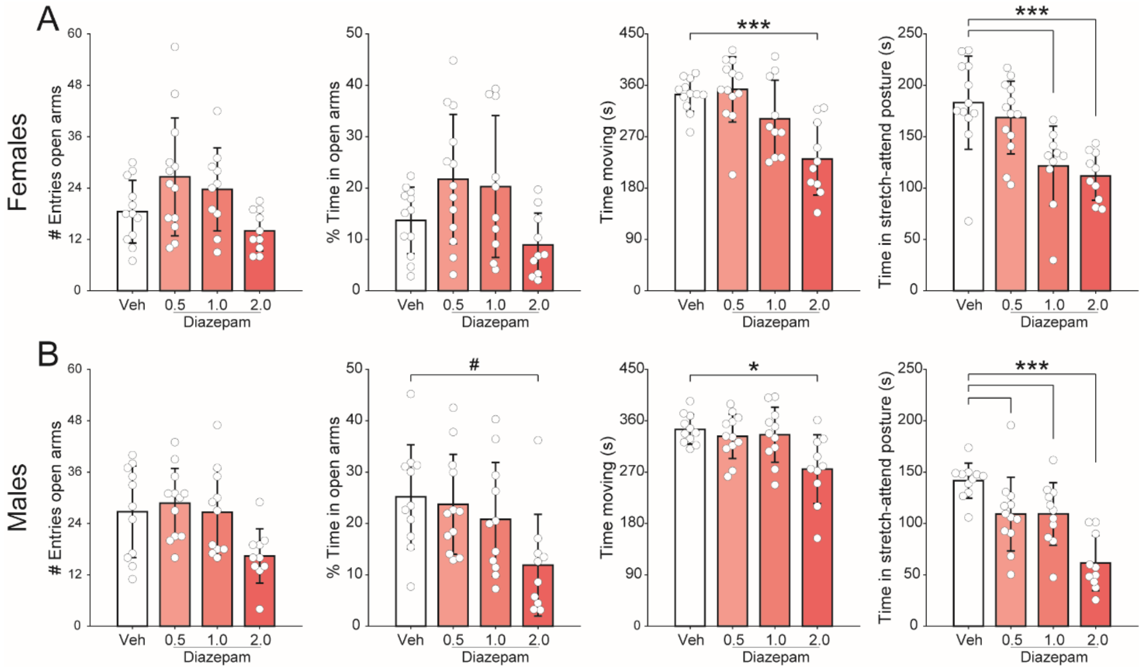

Figure S1. Elevated plus maze metrics for non-restrained female and male mice treated with diazepam. The columns respectively show the total number of entries in the open arms, total time spent in the open arms, total time moving, and total time spent in stretch-attend posture for the whole 10-min session shown separately for female (A) and male mice (B). The bars represent the mean (±SD) over animals. Notice, for both sexes, lack of anxiolytic-like effects of diazepam (first two columns) along with a decrease of locomotor activity (third column) and risk-assessment behavior shown as reduced time in stretch-attend posture (last column). $^{\#}p<0.05$, $^{*}p<0.01$, $^{***}p<0.0001$, one-way ANOVA followed by Tukey's post hoc test.

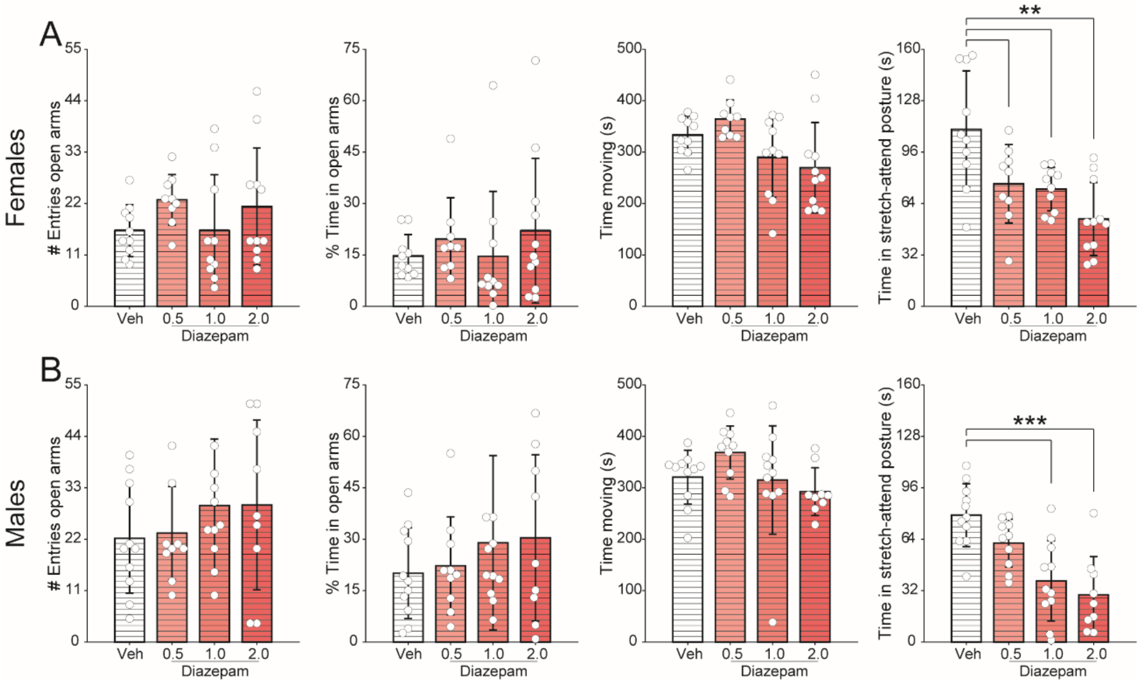

Figure S2. Elevated plus maze metrics for restrained female and male mice treated with diazepam. As in Figure S1, but for animals subjected to 1 hour of restraint-stress before the i.p. injection. The columns respectively show the total number of entries in the open arms, total time spent in the open arms, total time moving, and total time spent in stretch-attend posture for the whole 10-min session shown separately for female (A) and male mice (B). The bars represent the mean (±SD) over animals. Notice, for both sexes, lack of anxiolytic-like effects of diazepam (first two columns) along with a decrease of risk-assessment behavior shown as reduced time in stretch-attend posture (last column). **p<0.001, ***p<0.0001, one-way ANOVA followed by Tukey's post hoc test.

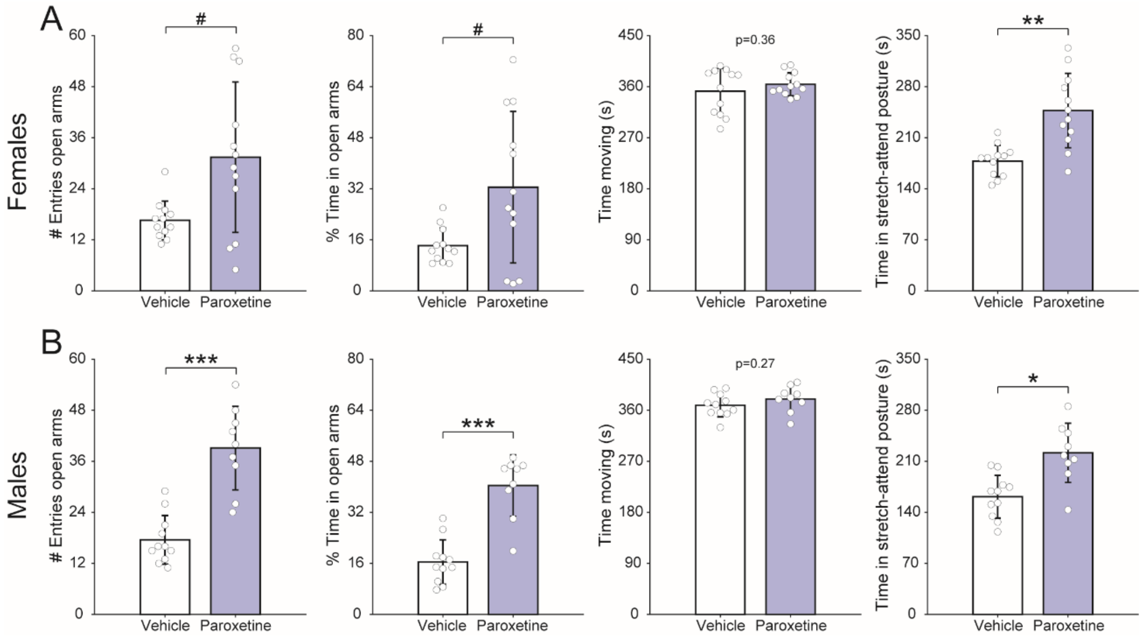

Figure S3. Anxiolytic-like action of paroxetine in female and male mice subjected to the elevated plus maze. As in Figure S1, but for animals treated with vehicle or paroxetine (10 mg/kg). The columns respectively show the total number of entries in the open arms, total time spent in the open arms, total time moving, and total time spent in stretch-attend posture for the whole 10-min session shown separately for female (A) and male mice (B). The bars represent the mean (±SD) over animals. Notice, for both sexes, anxiolytic-like effects of diazepam (first two columns) along with an increase of risk-assessment behavior shown as increased time in stretch-attend posture (last column). $^{\#}p<0.05$, $^{*}p<0.01$, $^{**}p<0.001$, $^{***}p<0.0001$, two-sample t-test.

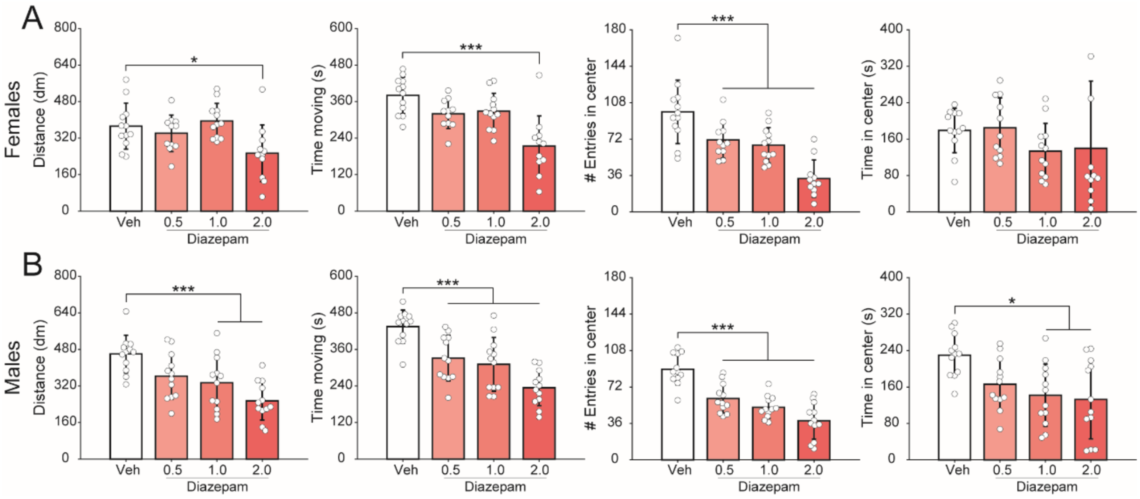

Figure S4. Diazepam effects in female and male mice in the open field. Distance traveled, total time moving, number of entries in the center zone, and time spent in the center of the open field for diazepam (0.5, 1 and 2 mg/kg) treated animals for the whole 10-min session shown separately for female (A) and male (B) mice. The bars represent the mean (±SD) over animals. Notice, for both sexes, that diazepam-treated animals display lower locomotor activity and entries into the center zone than vehicle-treated mice. *p<0.01, ***p<0.0001, one-way ANOVA followed by Tukey's post hoc test.

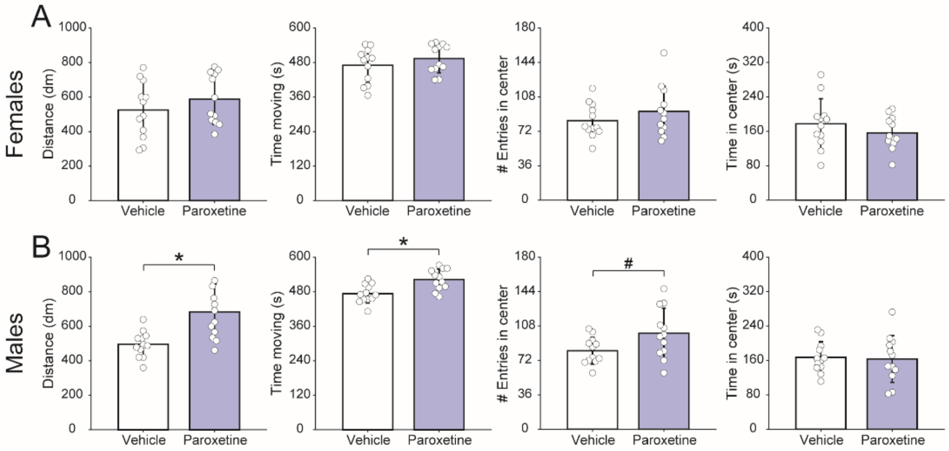

Figure S5. Paroxetine effects in female and male mice in the open field. Distance traveled, total time moving, number of entries in the center zone, and time spent in the center of the open field for paroxetine (10 mg/kg) treated animals for the whole 10-min session shown separately for female (A) and male (B) mice. The bars represent the mean (±SD) over animals. Notice higher activity for male mice treated with paroxetine. $^{\#}p<0.05$, $^{*}p<0.01$, two-sample t-test.
